# Programmed cell death-1 blockade in recurrent disseminated Ewing sarcoma

**DOI:** 10.1186/s13045-016-0278-x

**Published:** 2016-06-03

**Authors:** Georgia J. B. McCaughan, Michael J. Fulham, Annabelle Mahar, Judy Soper, Angela M. Hong, Paul D. Stalley, Martin H. N. Tattersall, Vivek A. Bhadri

**Affiliations:** Royal Prince Alfred Hospital, Camperdown, NSW Australia; Sydney Medical School, University of Sydney, Sydney, NSW Australia; Specialist Magnetic Resonance Imaging, Newton, NSW Australia; Chris O’Brien Lifehouse, 119-143 Missenden Road, Camperdown, NSW 2050 Australia

**Keywords:** Ewing sarcoma, Immunotherapy, Anti-programmed cell death-1 antibody, Case report

## Abstract

**Background:**

Ewing sarcoma (EWS) is a malignant tumour of bone and soft tissue, and although many patients are cured with conventional multimodal therapy, those with recurrent or metastatic disease have a poor prognosis. Genomic instability and programmed cell death ligand-1 (PD-L1) expression have been identified in EWS, providing a rationale for treatment with agents that block the programmed cell death-1 (PD-1) receptor.

**Case presentation:**

In this report, we describe a heavily pre-treated patient with recurrent metastatic EWS who achieved a clinical and radiological remission with PD-1 blockade.

**Conclusions:**

To our knowledge, this is the first reported case demonstrating efficacy of PD-1 blockade in EWS. This warrants further investigation in particular given the poor prognosis in patients with recurrent or metastatic disease.

## Background

Ewing sarcoma (EWS) is a poorly differentiated, aggressive malignant small blue round cell tumour of the bone and soft tissue [1]. It accounts for 34 % of primary bone tumours in people <20 years with an incidence of 2.9 per million [2]. Conventional treatment for localised disease is induction multi-agent chemotherapy then local control with surgery and/or radiation therapy followed by consolidation chemotherapy [3]. Nevertheless, approximately 30–40 % of patients develop recurrent local or metastatic disease, which is associated with a poor prognosis and a 5-year survival of 10–25 % [4–6].

Immune therapy using agents that target the programmed cell death-1 (PD-1) receptor interaction via its ligands PD-L1 or PD-L2 has shown efficacy in patients with advanced melanoma, non-small cell lung cancer, colorectal cancer, renal cell carcinoma, Hodgkin lymphoma and other malignancies [7–10]. In colorectal cancer, clinical response is correlated with mis-match repair (MMR) deficiency and microsatellite instability [8, 11]. Genomic instability has been described in EWS providing a rationale for treatment with immune checkpoint blockade [12, 13]. To our knowledge, the patient described here is the first to document the efficacy of PD-1 blockade in EWS.

## Case presentation

**Fig. 1:**
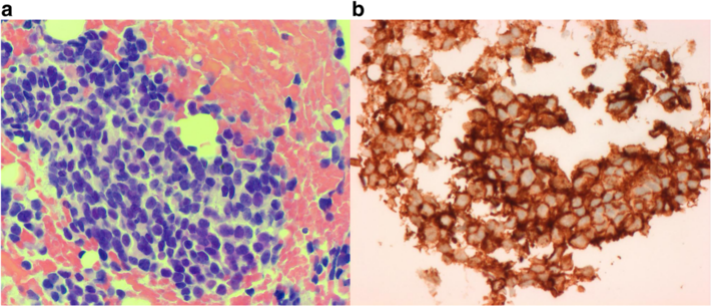
**a** Haematoxylin and eosin stain of primary tumour showing a small round blue cell tumour and **b** CD99 immunostain of primary tumour showing positive staining with a membranous pattern

Our patient is a 19-year-old male who presented in 2010 with left arm weakness secondary to a left C4-C6 paravertebral mass with bony involvement of C6 and T1. There were no additional sites of disease on a chest CT and a whole body ^18^F-FDG PET-CT scan. A CT-guided core biopsy showed a tumour composed of uniform small round cells with hyperchromatic round nuclei and scant cytoplasm (Fig. 1a). The neoplastic cells showed positive staining in immunostains for CD99 (membranous; Fig. 1b) and FLI-1 (nuclear) and were negative in immunostains for desmin, myogenin and S-100. Intracytoplasmic glycogen was demonstrated in a PAS stain. The combined morphological and immunophenotypic features were consistent with EWS (the presence of *EWSR1* rearrangement was confirmed on a subsequent biopsy). The patient received chemotherapy (vincristine/doxorubicin/cyclophosphamide and ifosfamide/etoposide) and local intensity modulated radiation therapy (55Gy/25#) to his mid-cervical spine. At the end of treatment, there was no clinical or radiological evidence of residual or recurrent disease.

Fifteen months after diagnosis, surveillance imaging identified bony and pulmonary metastatic disease. Biopsy of a right humeral lesion was morphologically consistent with recurrent EWS and molecular testing for the *EWSR1* rearrangement was positive. Over the next 4 years, he was treated with multiple chemotherapy regimens including irinotecan/temozolamide, high-dose ifosfamide, gemcitabine/docetaxel, a hedgehog signalling pathway inhibitor (LDE225) and carboplatin/etoposide. He had palliative radiotherapy to multiple bony sites including the right humerus, left ilium, thoracic and lumbar spine and bilateral whole lung radiation with additional stereotactic therapy to the largest pulmonary metastases. Over this period, there were short-lived responses and periods of stable disease but a clinical or radiological second remission was not achieved.

**Fig. 2:**
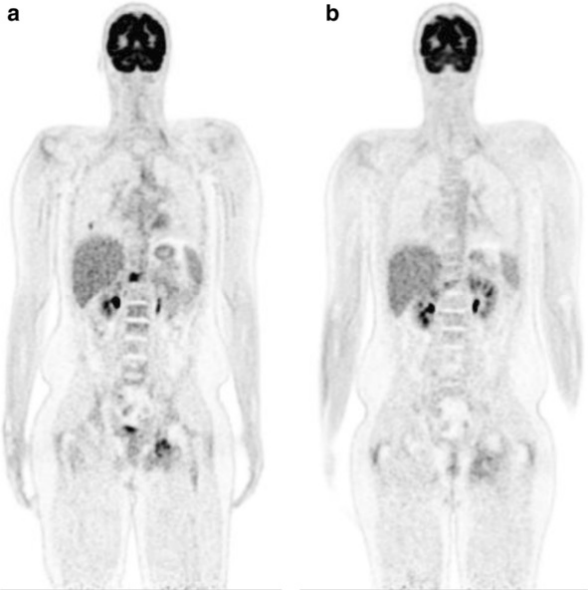
Coronal ^18^F-FDG PET-CT scans done prior to (**a**) and after (**b**) 3 cycles of pembrolizumab. The markedly increased FDG uptake in the right side and adjacent soft tissues of T12, in the left ischium and in one of the right middle lobe pulmonary metastases are shown. Post-treatment the FDG avidity in the bony lesions is much reduced and the right middle lobe lesion had completely resolved

**Fig. 3:**
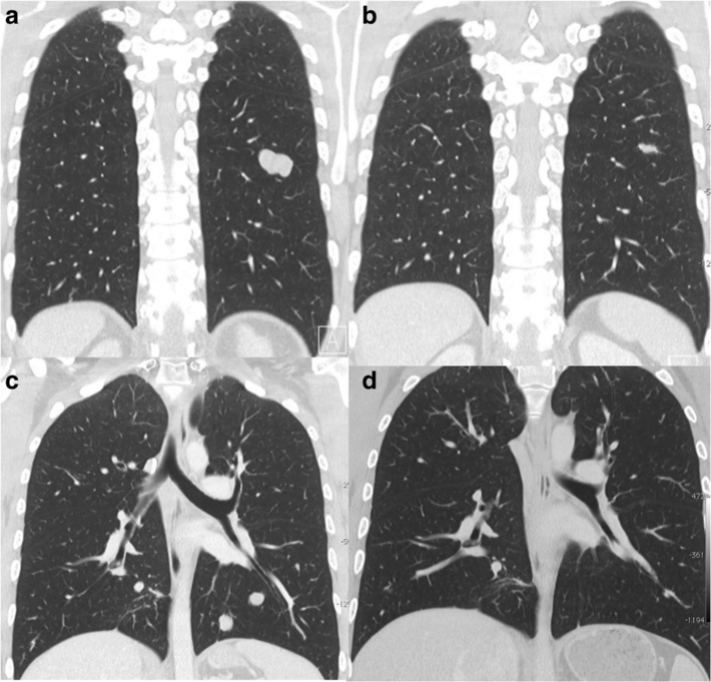
Coronal chest CTs done prior to (**a**, **c**) and after (**b**, **d**) 3 cycles of pembrolizumab show a marked decrease in size of the bilobed nodule in the superior segment of left lower lobe and complete resolution of the smaller left lower lobe nodules

**Fig. 4:**
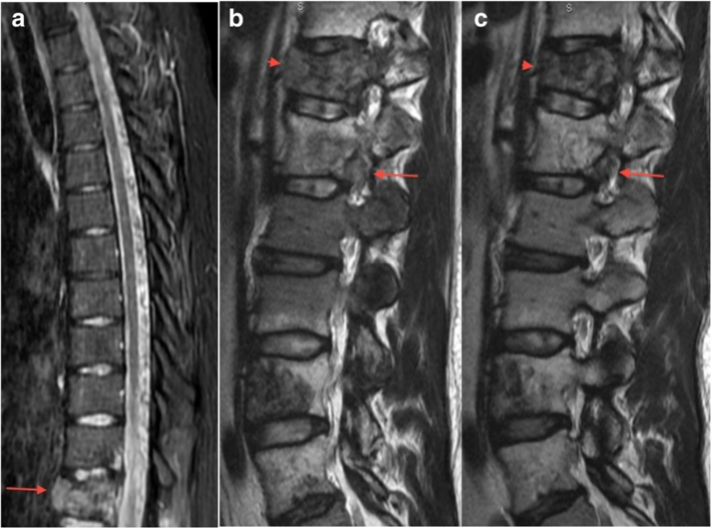
Sagittal MR images of thoracolumbar spine: **a** pre-treatment STIR demonstrates the lesion at T12 with extension through the anterior vertebral body bony margin; **b** pre-treatment T2 demonstrates tumour projecting into the T12 prevertebral soft tissue (*arrowhead*) and into the neural foramen at L1 (*long arrow*); and **c** post 3 cycles of pembrolizumab, there is no longer prevertebral extension of tumour at T12 (*arrowhead*) and only ill-defined soft tissue remains around the L1 root (*long arrow*); lesions in the body of L1, L4 and L5 are also smaller

In May 2015, just over 5 years from diagnosis, restaging whole body ^18^F-FDG PET-CT demonstrated multiple pulmonary metastases and increased FDG uptake at T11, T12 and the left ischium (Fig. 2a). The peak standardised uptake value (SUV) in the T12 lesion was 14.0. Chest CT confirmed 43 nodules of varying sizes throughout both lung fields (Fig. 3a, c) and thoracolumbar spine MR imaging demonstrated bony metastatic disease at T12, L1, L2, L4 and L5 with associated soft tissue mass at T12/L1 (Fig. 4a, b). He complained of low back pain but was otherwise asymptomatic with ECOG performance score of 0.

The patient commenced treatment with pembrolizumab (Keytruda, MSD) at 2 mg/kg intravenously every 3 weeks. The first cycle was complicated by fever without an identified source but there were no other immune-related adverse events. Restaging after cycle 3 showed a very good response to therapy with complete resolution of all but 4 of the pulmonary metastases. The largest nodule in the left lower lobe had reduced in diameter from 28 to 14 mm and peak SUV was 1.2 compared to 4.3 prior to treatment (Figs. 2b and 3b, d). The soft tissue component of the lesion at T12 had decreased in size and had a reduction in SUV from 14 to 6.1 (Figs. 2b and 4c). In addition, there was resolution of the soft tissue component anteriorly at L1, reduction in size of the lesion at L2 and better definition of the lesions at L4 and L5 (Fig. 4c). Clinically, his back pain resolved. After a further 6 cycles of pembrolizumab, progress imaging confirmed ongoing response to therapy, with complete resolution of active pulmonary metastases, a reduction in SUV at T12 from 6.1 to 4 and stable appearance on MR imaging (not shown). Treatment was ceased after a total of 9 cycles and at the most recent review 6 months since the last dose the clinical and radiological response has been sustained.

### Discussion

PD-L1 expression has been evaluated in a variety of sarcomas. The series reported by Raj et al. identified that 39 % of Ewing sarcomas expressed PD-L1 compared with 36 % of osteosarcomas and 97 % of leiomyosarcomas [14]. Kozak et al. retrospectively analysed 36 tumour samples of patients with synovial sarcoma and found PD-L1 expression in 83 %, with expression associated with poorer outcomes [15]. Kim et al. reported a similar association in a study of soft tissue sarcomas [16]. Of note, there is evidence across most, but not all, tumour types of a relationship between PD-L1 expression and response rates to immune checkpoint blockade, however a lack of PD-L1 expression does not preclude a response to PD-1 inhibition [7, 17–20]. Despite the known expression of PD-L1 in sarcoma, the only report of immune checkpoint blockade is a small pilot study by Maki et al. of the CTLA-4 inhibitor ipilimumab in advanced synovial sarcoma, in which no objective clinical responses were seen [21]. Another rationale for the possible utility of PD-1 blockade in EWS is the presence of genomic instability [12, 13]. Response to PD-1 blockade in colorectal cancer has been correlated with MMR deficiency; however, there are conflicting reports of MMR status and response in gastric cancer [8, 11, 22].

The explanation for the immune therapy response in our patient is unclear. Unfortunately, archival tissue was unavailable and hence we were unable to correlate response with PD-L1 expression (or lack of) in this case. His clinical course was relatively indolent for metastatic EWS and he had received extensive prior radiotherapy, which may have heightened the immune response. There is emerging evidence to suggest a role for radiotherapy in enhancing responses to immune therapy through a variety of mechanisms [23, 24].

## Conclusions

To our knowledge, this is the first reported case of response of EWS to immune checkpoint blockade with pembrolizumab. The patient’s recurrent metastatic EWS responded poorly to multiple conventional and experimental chemotherapeutic agents over the preceding 4 years, and his early relapse is a well-recognised poor prognostic marker in recurrent EWS [4–6, 25]. We suggest that further evaluation of PD-1-directed therapy in recurrent and metastatic EWS is warranted and await the results of the SARC028 phase II study investigating efficacy and safety of pembrolizumab in advanced soft tissue and bone sarcomas [26]. In addition, evaluation of pre-treatment and on-treatment biomarkers in SARC028 and future studies will refine our ability to predict response to immune checkpoint blockade in individual patients and monitor treatment efficacy [20, 26].

## Abbreviations

EWS: Ewing sarcoma
MMR: mis-match repair
PD-1: programmed cell death-1
PD-L1/2: programmed cell death ligand-1/2
SUV: standardised uptake value
